# Semantic-Guided Adaptive Gaussian Segmentation

**DOI:** 10.3390/s26196173

**Published:** 2026-09-29

**Authors:** Yinghan Zhou, Fan Zhou

**Affiliations:** College of Information Engineering, Shanghai Maritime University, Shanghai 201306, China; 202310310201@stu.shmtu.edu.cn

**Keywords:** 3D gaussian splatting, 3D scene segmentation, semantic guidance, multi-view reconstruction, 3D consistency, gaussian decomposition, feature fusion

## Abstract

3D Gaussian Splatting (3DGS) enables real-time photorealistic scene reconstruction, yet its segmentation tasks suffer from two critical flaws: poor 3D consistency (e.g., blurred instance boundaries and unstable cross-view semantic association) and insufficient structural awareness near ambiguous object boundaries. To address these issues, this paper proposes a semantic-guided 3D Gaussian segmentation framework. We first process multi-view RGB images and semantic masks to generate 2D semantic codes and initial Gaussian parameters. We then build a Gaussian-level joint representation by combining flow-aligned appearance cues, CLIP-derived semantic descriptors, and explicit Gaussian geometry. Semantic-guided adaptive decomposition identifies boundary-sensitive Gaussians through cross-view boundary statistics and semantic uncertainty, while multi-view voting further refines instance boundaries. Finally, 3D global optimization unifies instance association and labeling. Experiments on ScanNet and SPIn-NeRF show improved results under the reported evaluation protocol and the stated subset definitions. In particular, on the ScanNet benchmark, compared with the evaluated 3DGS baseline InstanceGaussian, SG-AGS improves mAcc@0.25 by 16.2 points for category-agnostic 3D instance segmentation and by 25.6 points for text-query-based open-vocabulary labeling of segmented 3D instances. These results support the usefulness of SG-AGS on the two evaluated benchmarks and suggest potential value for object-level scene querying and digital-twin inspection, while broader generalization to external captures and other scene domains still requires further validation.

## 1. Introduction

3D scene segmentation aims to decompose spatial structures into semantically meaningful instances and is a common task in scene understanding scenarios such as autonomous driving, augmented reality (AR), and robotic manipulation. Traditional approaches based on LiDAR point clouds or multi-view stereo (MVS) meshes can be less convenient in settings that rely on standard RGB capture and photorealistic rendering pipelines. This problem requires not only accurate geometric reconstruction but also consistent semantic understanding across multiple views.

Neural Radiance Fields (NeRF) introduced a new way of modeling 3D scenes through implicit neural representations, enabling joint reconstruction and segmentation without explicitly defined geometry. However, its volumetric rendering process is computationally expensive, and real-time performance is still difficult to achieve in many scenarios [1]. To alleviate this issue, 3DGS has recently emerged as a more efficient alternative. By representing scenes as a set of learnable 3D Gaussians with explicit spatial and appearance parameters, 3DGS enables high-quality rendering at real-time frame rates, and has gradually become a common choice for modern reconstruction systems.

Despite its success in rendering, applying 3DGS directly to segmentation tasks is still not straightforward. Existing methods often face two main limitations. First, there is a lack of strong cross-view consistency. Many approaches rely on projected language features or local feature grouping strategies [2,3], which may not fully capture global geometric and semantic relationships. As a result, the same physical region can sometimes receive inconsistent labels across different views, and object boundaries may appear less stable, especially in cluttered or thin structures [4]. Second, current segmentation pipelines built on 3DGS are not always optimized from a structural perspective. Even when reconstruction itself is efficient, Gaussian distributions are often not explicitly controlled for segmentation purposes, which can leave mixed primitives near ambiguous object boundaries and weaken the clarity of instance separation.

To address these issues, we propose SG-AGS, a 3DGS-based instance segmentation framework designed for text-query-based labeling of segmented 3D instances. Different from pipelines that only aggregate 2D semantics onto fixed 3DGS primitives or trigger Gaussian refinement mainly from geometry-dominant heuristics, our method uses 2D semantic evidence as a boundary diagnosis signal, verifies the actual split through cross-view boundary statistics and voting confidence, and then reconnects the updated primitives through graph-based consistency propagation. In our implementation, these 2D semantic cues are introduced through precomputed masks, semantic descriptors [5], and optical-flow alignment, which allows the model to support flexible text-query labeling of segmented 3D instances while keeping the semantic pipeline and the reconstruction pipeline clearly separated.

In practice, we observe that boundary regions are often not well-optimized under standard training procedures [6]. Some Gaussians near object transitions tend to remain ambiguous or under-refined. Instead of directly assigning fixed labels, we design a decomposition mechanism in which 2D semantic inconsistency serves only as a candidate-detection signal. The final decomposition decision is made after combining cross-view boundary-hit statistics and multi-view semantic uncertainty, so that isolated mask noise or a single unstable view does not directly trigger splitting. Only after this verification step do we decompose the Gaussian along its principal geometric direction. This process helps refine local structure while keeping the geometric interpretation of the split physically grounded.

In addition, we introduce a unified feature representation that jointly encodes appearance, semantic cues, and geometric attributes such as scale and rotation. This representation is used as a lightweight Gaussian-level fusion descriptor for voting, decomposition, and graph propagation, rather than as an independently pre-trained semantic classifier. Although ambiguity may still remain in challenging cases, such as highly similar objects, the unified descriptor makes later semantic decisions more stable across views. Furthermore, we build a global consistency optimization module using a k-nearest neighbor graph, where spatial proximity and feature similarity are both considered. Through this mechanism, label consistency across the scene is encouraged, and isolated misclassifications can be partially corrected.

The main contributions of this work can be summarized as follows:

- We present a semantic-guided Gaussian decomposition strategy within the 3DGS framework, which uses 2D semantic evidence to diagnose candidate boundary ambiguity, verifies the split through cross-view boundary statistics and voting confidence, and then performs the actual split along the principal direction of the 3D covariance.
- We design a unified feature encoding scheme that integrates geometric, appearance, and semantic cues into a shared representation space, improving instance-level feature discriminability.
- We propose a global consistency optimization strategy based on k-nearest neighbor graph propagation, which helps improve cross-view coherence and reduces local labeling inconsistencies.
- We report results on standard 3D instance segmentation benchmarks and analyze overall segmentation accuracy under the stated evaluation protocols, while discussing boundary quality and semantic consistency through the reported qualitative examples and cross-view verification results.

## 2. Related Works

Recent years have seen continued progress in novel view synthesis, largely driven by Neural Radiance Fields (NeRF). By modeling scenes as continuous volumetric radiance fields, NeRF achieves high-quality rendering. However, NeRF and its successors rely heavily on volumetric ray marching to accumulate density and color, which introduces computational overhead for interactive applications. 3DGS provides an explicit alternative by parameterizing appearance and spatial geometry with anisotropic 3D Gaussian primitives. Paired with a tile-based rasterizer, it supports efficient rendering and has become a common choice for 3D reconstruction. A key limitation lies in its native formulation: standard 3DGS is designed primarily to fit low-level photometric signals such as color and opacity, while its discrete primitives are distributed in space without inherent semantic structure [7]. This absence of high-level awareness makes it difficult to directly apply vanilla 3DGS to tasks requiring semantic reasoning or instance-level manipulation, such as augmented reality or autonomous driving [8].

As vision-language models (VLMs) such as SAM and CLIP continue to demonstrate effective 2D zero-shot recognition [9], researchers have sought to distill these 2D priors into 3D environments. Several 3DGS-based frameworks, such as GaussianGrouping, Feature 3DGS, and LangSplat, attach high-dimensional mask features or language embeddings to Gaussian primitives. This enables text-driven queries within reconstructed scenes. However, these methods can still suffer from view-dependent bias. Because their semantic information is typically derived from 2D predictions and projected into 3D space, the resulting semantics may be weakly coupled with the actual geometry. Under occlusion or large viewpoint changes, these approaches may assign conflicting semantic labels to the same physical object [10], which can lead to blurred instance boundaries and cross-view semantic misalignment. Methods such as CaRF [11] enhance multi-view consistency in referring segmentation by incorporating camera geometry, but physical separation at instance boundaries under sparse or noisy observations remains challenging.

Beyond maintaining consistency across views, the effectiveness of a 3D segmentation pipeline depends on how it fuses multi-modal signals—specifically geometry [12], appearance, and high-level semantics. When lifting 2D foundation models into 3D representations [3], many existing scene understanding techniques adopt relatively straightforward strategies. They typically attach high-dimensional CLIP or SAM vectors onto individual Gaussians or rely on basic concatenation to build joint feature fields. While computationally efficient, such fusion may not fully leverage the physical properties of the scene. 3DGS encodes local geometric parameters, such as covariance and scale [13], which often correlate with semantic structures (e.g., a flat tabletop versus a thin pole). By relying primarily on shallow feature concatenation and bypassing these geometric cues, current methods can produce embeddings that are less structured for reliable instance clustering in dense 3D spaces [14].

Furthermore, the boundary fidelity [15] of instance-level segmentation depends on how the underlying Gaussians are managed. The original 3DGS framework uses a heuristic density control strategy, cloning or splitting primitives based on view-space positional gradients. Newer methods have introduced Gaussian pruning and lightweight encoding techniques (e.g., EAGLES [16]), which are primarily guided by volumetric density or visual saliency. However, these optimization schemes operate largely independently of semantic boundaries. Because they are driven primarily by geometric cues or photometric errors, they may still leave mixed Gaussians near ambiguous object borders and may not split Gaussians that straddle two distinct objects. As a result, mixed-semantic Gaussians can remain at physical boundaries, causing geometric under-segmentation and reducing the sharpness of extracted instances.

Recent frameworks such as InstanceGaussian extend instance-level understanding within 3DGS by projecting 2D masks for local feature grouping and refining Gaussian-level feature organization. These designs still involve trade-offs, and they also clarify more precisely where our mechanism differs. InstanceGaussian mainly improves Gaussian-level grouping after 2D mask projection, but it does not explicitly verify whether a mixed primitive should be decomposed through persistent cross-view semantic ambiguity [3]. Geometry-driven techniques such as SAGD and related splitting-based variants improve boundary fidelity through stochastic or geometry-dominant decomposition, yet their splitting criteria are not formulated around multi-view voting confidence derived from semantic aggregation [17,18]. In contrast, our method does not claim an entirely new decomposition family; rather, it combines four specific steps within one pipeline: candidate diagnosis from 2D semantic evidence, cross-view verification using boundary-hit statistics and voting confidence, joint-feature recomputation after decomposition, and k-NN graph propagation for global label correction. We therefore position SG-AGS as a semantic-guided integration of decomposition, voting, and graph refinement within 3DGS, instead of claiming universal superiority over every boundary-aware Gaussian splitting variant reported under different settings.

**Figure 1:**
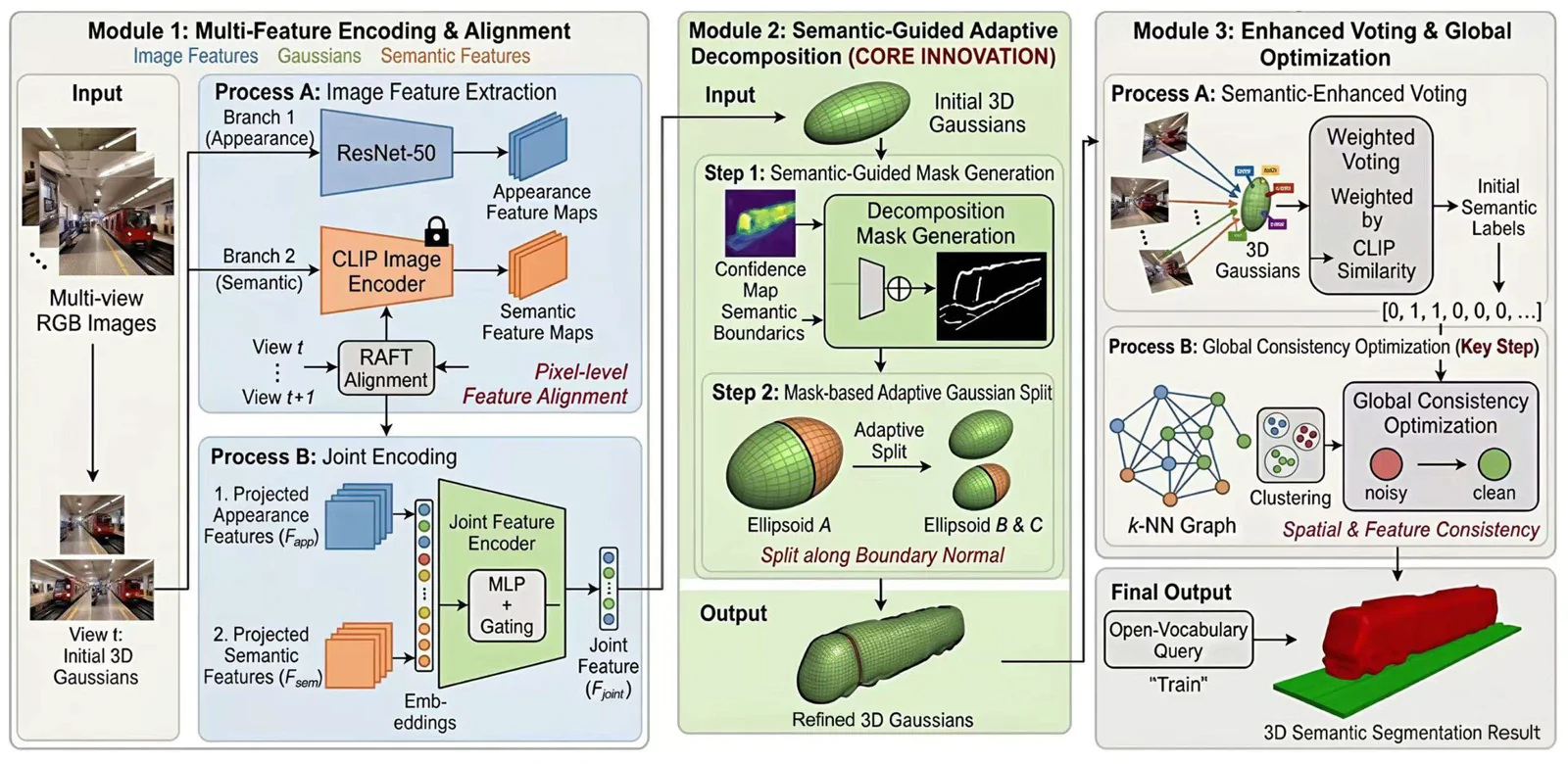
Pipeline of our proposed method. (1) Multi-Feature Encoding & Alignment Module: It extracts appearance and semantic features from multi-view RGB images and initial 3D Gaussians, aligns them at pixel level, and fuses them into a unified joint feature representation. (2) Semantic-Guided Adaptive Decomposition Module: It generates semantic-guided decomposition masks, uses 2D semantic inconsistency to identify boundary-sensitive candidates, and then splits the corresponding 3D Gaussians along the principal direction of their 3D covariance to produce refined, boundary-aligned primitives. (3) Enhanced Voting & Global Optimization Module: It performs semantic-enhanced weighted voting on refined Gaussians and conducts global consistency optimization to generate the final 3D semantic segmentation results.

Furthermore, the quality of the underlying 3D representation is tied to the robustness of the 2D visual inputs. Recent studies have shown that 2D preprocessing can improve downstream 3D modeling. For example, Vrochidis et al. [19] showed that colormap selection and image enhancement strategies can increase keypoint matching and reconstruction accuracy in photogrammetry. Inspired by such appearance-aware preprocessing insights, our framework emphasizes feature preprocessing by using frozen pre-trained models and optical flow alignment before geometric decomposition. An overview of the complete SG-AGS pipeline is shown in Figure 1, where the three blocks correspond to multi-feature alignment, semantic-guided adaptive decomposition, and global consistency refinement.

## 3. Research Methodology

### 3.1. Feature Alignment and Joint Feature Encoding

**Figure 2:**
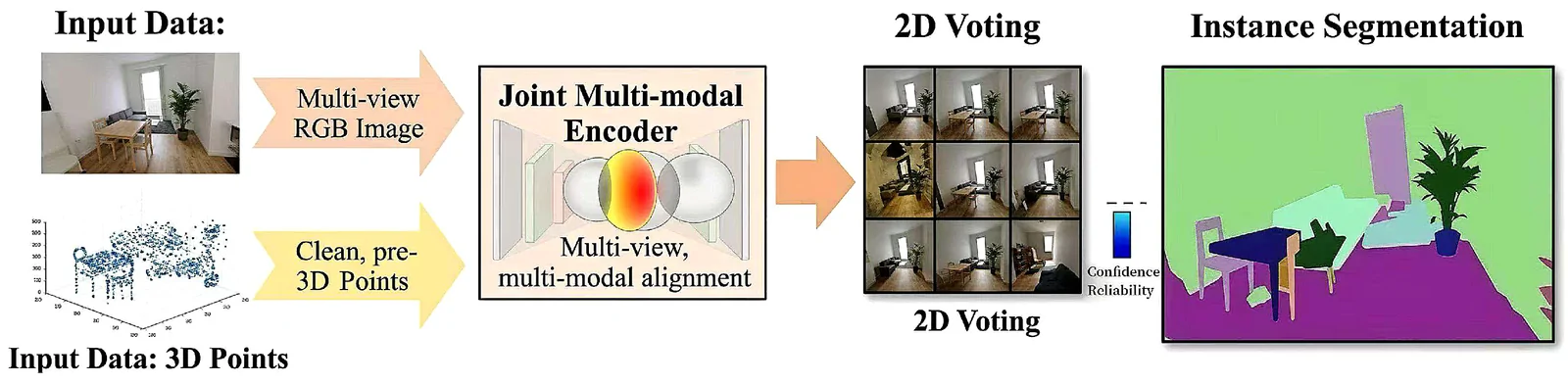
Overview of our Semantic Gaussians. We inject semantic features into off-the-shelf 3D Gaussian Splatting via multi-Modal Fusion and 2D voting.

To tackle the first issue of multi-view misalignment, we introduce an optical flow-guided spatial warping mechanism. When aggregating appearance features from multiple camera views onto a single 3D Gaussian, raw 2D projections often suffer from parallax errors, resulting in blurred feature fields. Instead of blindly averaging these features, we designate a reference viewpoint and compute dense optical flow fields via RAFT [20] for all other source views. The source feature maps are warped according to the estimated flow before projection, which improves pixel-level correspondence under moderate camera motion. Once aligned, the final appearance feature $f_{i}^{app}$ for a given Gaussian $g_{i}$ is computed through a distance-weighted average of the valid visible viewpoints. This explicit alignment reduces view-dependent feature drift and helps preserve local visual semantics near object boundaries. Figure 2 summarizes this semantic-Gaussian view at a high level: 2D semantic cues are injected into the Gaussian representation through multi-modal fusion and voting, and the enriched Gaussians are then used by the later decomposition and consistency modules. In the current system, RAFT serves as a practical appearance-alignment module before feature aggregation, rather than as a geometry-aware reprojection substitute; severe occlusion, very large viewpoint changes, or low-texture correspondences can still reduce its reliability.

However, resolving 2D visual misalignment is only part of the problem; robust instance-level understanding also requires 3D geometric awareness. The defining advantage of 3DGS is its explicit parameterization of local shape: each Gaussian $g_{i}$ possesses a 3-dimensional scale vector $s_{i}\in R^{3}$ and a 4-dimensional rotation quaternion $q_{i}\in R^{4}$. Together, these parameters define the 3D covariance matrix $\Sigma_{i}$, indicating whether a primitive is a flat disk, an elongated needle, or a uniform sphere. This explicit geometry provides a useful physical prior. To use this information, we design a lightweight Joint Feature Encoder. Rather than treating appearance, semantics, and geometry as isolated signals, this module normalizes and projects the three modalities into a shared representation used by clustering, voting, and graph propagation.

Specifically, the encoder ingests three distinct modalities. Let $f_{i}^{app}\in R^{256}$ denote the flow-aligned local appearance feature, $f_{i}^{sem}\in R^{512}$ denote the global CLIP-based semantic embedding, and $f_{i}^{geo}=\left[s_{i}\;\|\;q_{i}\right]\in R^{7}$ denote the explicit geometric vector constructed by concatenating the scale and rotation parameters. The operator ‖ denotes concatenation along the feature channel dimension. The joint feature $f_{i}^{\mathrm{joint}}\in R^{512}$ is computed via a non-linear projection:

$$f_{i}^{\mathrm{joint}}=E_{\mathrm{joint}}\left(f_{i}^{app}\left\|f_{i}^{sem}\right\|f_{i}^{geo}\right),$$

where $E_{\mathrm{joint}}$ represents the Joint Feature Encoder, implemented as a LayerNorm layer followed by a two-layer MLP with a 512-dimensional hidden layer and GELU activation. The encoder is used as a fusion and projection block rather than as a separately pre-trained classifier. In the current implementation, this block is parameterized as a standard LayerNorm+MLP projector, but we do not introduce a separate supervision objective, external pre-trained checkpoint, or standalone optimization stage for it. Instead, the appearance, semantic, and optical-flow cues are extracted from precomputed or fixed 2D sources, and the encoder is used only as a lightweight nonlinear fusion map that places appearance, semantic, and geometric descriptors into a shared 512-dimensional space before later clustering, boundary decomposition, and graph propagation. Accordingly, we do not interpret $f_{i}^{\mathrm{joint}}$ as an independently calibrated semantic embedding. Its practical role is more limited: it reduces scale mismatch across heterogeneous cues and provides a compact descriptor for within-scene comparison, while the later semantic decisions remain anchored to projected 2D mask labels, multi-view voting confidence, and spatial graph constraints rather than to this projector alone.

### 3.2. Semantic-Guided Adaptive Gaussian Decomposition

We introduce a semantic-guided adaptive decomposition mechanism [17,18] to resolve geometric under-segmentation. Rather than relying on random splitting, this approach first uses 2D semantic priors only to detect candidate boundary-crossing Gaussians, and then confirms whether they should be decomposed through cross-view consistency checks before fracturing them along their principal geometric axes.

To locate primitives that are likely to require splitting, we employ morphological gradient-based boundary detection. Given a 2D instance mask from any camera viewpoint, we calculate its morphological gradient to isolate semantic edge pixels. When we project the 3D Gaussians onto this view, we track how often each Gaussian’s center lands within these edge regions. In the current implementation, this center-based boundary hit is used only as a coarse pre-screening signal rather than as a full description of the projected Gaussian footprint. To avoid excessive splitting, we filter these candidates using confidence scores from our multi-view voting module. If a Gaussian frequently hits semantic boundaries across multiple views and simultaneously exhibits low voting confidence, it is marked as a decomposition candidate. In our design, this stage is intentionally conservative: a single inconsistent 2D observation is treated only as a heuristic signal, whereas actual decomposition is reserved for Gaussians whose boundary ambiguity persists under cross-view statistics. This produces a binary decomposition mask that focuses computation on primitives associated with boundary ambiguity and reduces unnecessary splitting in uniform regions [21].

Once a Gaussian is flagged as critical, we must verify if it is genuinely bridging two objects and determine exactly how to cut it. We analyze the 2D projected covariance matrix of the Gaussian, which mathematically describes its elliptical footprint on the image plane. By performing eigenvalue decomposition:

$$\Sigma_{i,k}^{2D}V=\Lambda V,$$

where $\Sigma_{i,k}^{2D}\in R^{2\times 2}$ is the 2D projected covariance matrix of Gaussian $g_{i}$ under view $v_{k}$, $V=[v_{1},v_{2}]$ is the matrix of orthogonal eigenvectors, and $\Lambda =\mathrm{diag}(\lambda_{1},\lambda_{2})$ is the diagonal matrix of corresponding eigenvalues with $\lambda_{1}\geq \lambda_{2}$.

We extract the principal eigenvector corresponding to the largest eigenvalue. Physically, this eigenvector represents the direction in which the Gaussian is most stretched. We then construct a symmetric probing segment centered at the projected mean $\mu_{i,k}^{2D}$ along the principal direction $v_{1}$. Let the total length of this segment be $3\sqrt{\lambda_{1}}$, and define its two endpoints as

$$p_{1,2}=\mu_{i,k}^{2D}\pm \frac{3}{2}\sqrt{\lambda_{1}}\;v_{1}.$$

where $p_{1}$ and $p_{2}$ represent the 2D pixel coordinates of the two endpoints, $\mu_{i,k}^{2D}\in R^{2}$ denotes the projected 2D center of Gaussian $g_{i}$ under view $v_{k}$, $\lambda_{1}$ is the largest eigenvalue, and $v_{1}\in R^{2}$ is the unit principal eigenvector. The scalar coefficient $\frac{3}{2}$ is applied such that the total length of the probing segment is $3\sqrt{\lambda_{1}}$. These two points are used to probe the semantic variation of the Gaussian support along its dominant projected axis. If the semantic labels at the two endpoints are different, the Gaussian is regarded as a boundary-sensitive candidate under the current view.

Next, we check the semantic labels of these two extreme points on the 2D mask. If the label at one end differs from the other, this serves as a strong heuristic indicator that the Gaussian may span two objects. To ensure robustness against 2D mask noise, occlusions, and depth ambiguity, we introduce a strict dual-threshold verification mechanism. A Gaussian is only treated as a valid decomposition candidate if it simultaneously satisfies a high boundary hit rate ($\tau_{\mathrm{bound}}>0.3$) across multiple views and exhibits a low multi-view voting confidence ($\tau_{\mathrm{conf}}<0.5$). In this way, the endpoint-label inconsistency is used as a diagnosis signal rather than as a direct splitting command. The actual decomposition is triggered only when this local inconsistency is repeatedly supported by cross-view evidence, which makes spurious splits under transient projection errors or isolated single-view occlusions much less likely.

To transfer the 2D diagnostic into a 3D decomposition direction, we perform eigenvalue decomposition directly on the true 3D covariance matrix to extract its 3D principal axis. We then project this 3D principal axis onto the 2D image plane and compute its dot product with the 2D semantic shift direction. The sign of this dot product determines the positive or negative spatial offset along the 3D principal axis. Therefore, our method does not assume that the dominant axis of the projected 2D covariance is strictly identical to the projected direction of the 3D principal axis under perspective projection. The 2D cue is used only for boundary diagnosis and sign disambiguation, whereas the actual split axis remains defined by the 3D covariance itself. This design reduces, but does not completely eliminate, directional inconsistency under strong perspective distortion. In particular, when a Gaussian becomes nearly isotropic or when the leading eigenvalues are very close, the directional sign estimated from the 2D diagnostic may still become unstable after projection. In the current implementation, we do not introduce an additional eigen-gap-based fallback rule for such cases; instead, we mitigate them conservatively through multi-view boundary-hit verification, low-confidence filtering, and localized scale attenuation after splitting. To avoid creating unstable small splats, the splitting ratio is adjusted according to the proportion of the projected support that falls into each semantic region. During decomposition, the child Gaussians inherit the parent’s opacity, rotation, and spherical-harmonic coefficients as an initialization, while their centers are symmetrically displaced along the principal axis. Their scales are reduced according to the estimated split ratio so that the two child primitives remain locally concentrated after the split. Accordingly, the covariance of each child Gaussian is not directly inherited as a fixed matrix, but is re-parameterized from the updated scale and the inherited rotation. After decomposition, the Gaussian-level joint features are recomputed from the updated geometry together with the re-aggregated appearance and semantic cues in the subsequent feature extraction stage. This covariance-driven splitting [22] reduces the number of mixed-semantic primitives near object boundaries before global clustering, while the remaining near-isotropic failure cases are treated as a current limitation rather than as fully solved cases.

### 3.3. Semantic-Enhanced Voting and Global Consistency Optimization

To address cross-view label conflicts and ensure globally consistent instance IDs, we introduce a semantic-enhanced adaptive voting mechanism backed by a global 3D consistency graph.

After encoding the joint features and splitting boundary-sensitive Gaussians, the next step is assigning an instance label to every fine-grained primitive. Traditional multi-view backprojection methods typically use simple majority voting. They count how many times a Gaussian falls inside a specific 2D mask across different camera angles and select the most frequent label. This unweighted approach is sensitive to occluded, blurry, or mis-segmented views because every view contributes equally. As a result, local label fragmentation can appear near occluded object boundaries. To reduce this inconsistency, we introduce a semantic-enhanced adaptive voting mechanism backed by a global 3D consistency graph.

During the initial voting phase, we project all primitives onto the training views to sample their 2D mask labels [23]. However, instead of treating every view equally, we weight each vote using the joint features generated earlier. Specifically, the algorithm calculates the cosine similarity between a Gaussian’s joint feature and the average feature of the 2D mask region it falls into. In the current implementation, this similarity is used only as a relative within-scene weighting cue after the Gaussian has already been associated with a visible 2D region by projection; it is not treated as a standalone semantic classifier. A higher similarity therefore indicates better local agreement between the fused Gaussian descriptor and the projected region under the current scene and view, so the corresponding vote receives a larger weight. We also apply a preference multiplier when the 2D region exhibits a strong CLIP language response. Boundary Gaussians produced by the decomposition stage are assigned additional voting weight because they are more likely to lie near ambiguous instance transitions. Once the multi-view aggregation concludes, every Gaussian receives a preliminary instance label alongside a cumulative confidence score.

Although weighted voting reduces single-view noise, large-scale scenes are rarely observed uniformly. Some Gaussians may remain weakly observed or have low voting confidence because of occlusion and sparse views. To regularize these uncertain primitives, we build a k-nearest neighbor (k-NN) global consistency graph based on dual spatial and feature constraints. In the current implementation, each Gaussian is connected to its k=12 nearest neighbors in 3D center space. The edge weight between $g_{i}$ and $g_{j}$ is defined as

$$w_{ij}=\exp\left(-\frac{{∥\mu_{i}-\mu_{j}∥}_{2}^{2}}{2\sigma_{s}^{2}}\right)\cdot \frac{1+\cos(f_{i}^{\mathrm{joint}},f_{j}^{\mathrm{joint}})}{2},$$

where $\mu_{i}$ and $\mu_{j}$ denote Gaussian centers, $\sigma_{s}$ is set to the median neighbor distance in each scene, and the cosine term is normalized to [0, 1]. This normalization reduces sensitivity to scene scale and prevents negative cosine similarities from producing invalid edge weights. Strong graph connections are therefore formed only between Gaussians that are spatially close and similar in the joint feature space.

With this global graph established, we execute a confidence-driven label smoothing protocol to improve consistency across the scene. When a node has low voting confidence, the algorithm checks its highest-weight graph neighbors. If these neighbors have similar joint features and agree on a specific instance label, the low-confidence node is reassigned to the local consensus label. Iterating this process reduces local conflicts introduced by uncertain viewpoints and encourages Gaussian elements from the same physical entity to maintain spatial continuity and semantic coherence. Ultimately, the system pools the dominant CLIP features within each refined cluster, outputting a 3D instance ID that is later used for text-query-based category assignment [24].

## 4. Results

### 4.1. Experimental Setup

#### 4.1.1. Dataset: SPIn-NeRF

The full SPIn-NeRF benchmark contains 10 real-world scenes captured by calibrated multi-view cameras, covering complex geometric structures and diverse material properties, which makes it challenging for both 3D reconstruction and segmentation. The scenes include fine-grained structures with severe occlusion and high curvature variation, as well as non-Lambertian surfaces that produce strong view-dependent appearance changes. Moreover, SPIn-NeRF follows a sparse-view setting, where each scene typically provides only 20–60 input images, imposing stringent requirements on cross-view geometric and semantic consistency. In our quantitative comparison, the full SPIn-NeRF benchmark contains 10 scenes, while Table 1 lists the eight scenes for which scene-wise comparisons are reported in this manuscript, namely Otters, Ferns, Room, Fork, Fortress, Bark, Truck, and Lego. Among them, Bark is kept explicitly as a failure case for SG-AGS. For the summary statistics, we use the common seven-scene valid subset (Otters, Ferns, Room, Fork, Fortress, Truck, and Lego), on which valid results are available for all compared methods under the stated evaluation protocol. For evaluation, we use the provided 2D object masks as ground truth, compare the rendered predictions against the corresponding view-wise masks in a pixel-wise manner, and report Accuracy and Intersection over Union (IoU) as the main quantitative metrics. This benchmark mainly reflects sparse-view multi-view reconstruction scenes with challenging boundaries and viewpoint changes; by itself, it does not establish broader cross-domain generalization.

#### 4.1.2. Dataset: ScanNet

The ScanNet dataset [25] is a large-scale indoor RGB-D benchmark that contains over 1500 real-world scenes reconstructed from posed RGB-D sequences. Each scene provides densely sampled viewpoints with high-resolution RGB images, depth maps, and accurate camera poses, along with pixel-level semantic and instance annotations. Compared to SPIn-NeRF, ScanNet presents a complementary evaluation setting with significantly denser view coverage and more diverse indoor layouts. The dataset includes a wide variety of object categories (e.g., chairs, tables, cabinets) arranged in cluttered environments, featuring frequent object occlusions and complex spatial relationships. Such characteristics make it particularly suitable for evaluating the robustness of 3D instance segmentation methods in real-world indoor scenarios. For our experiments, we follow standard preprocessing protocols and utilize the provided RGB images and camera poses to construct the 3D Gaussian representation. Ground-truth segmentation masks are obtained from the annotated 2D labels, which are projected into each view for evaluation. We perform pixel-wise comparisons between predicted segmentation results and ground-truth masks across multiple viewpoints, and report standard metrics including Accuracy and Intersection over Union (IoU). Together, ScanNet and SPIn-NeRF provide complementary indoor and sparse-view evaluations in this study, but they do not cover all external real-world settings or scene types.

#### 4.1.3. Implementation Details

To construct high-fidelity 3D scenes and support reproducibility, the underlying reconstruction framework in this study follows the standard pipeline of 3DGS [23] and is implemented under the PyTorch framework. All experiments are conducted on a single NVIDIA vGPU equipped with 32 GB of VRAM. Given the input multi-view image sequences, we first utilize COLMAP to extract an initial sparse point cloud as the initialization of 3D Gaussian centers, which provides a reliable geometric prior for subsequent geometric optimization. In the model training phase, we adopt the Adam optimizer and set the total number of iterations for the entire reconstruction process to 30,000 uniformly. To balance rapid convergence and fine-grained geometry, we use a fixed learning rate schedule: the learning rate for the spatial position of Gaussian primitives is initialized at $1.6\times 10^{-4}$ and decays exponentially to $1.6\times 10^{-6}$ with iterations; the learning rates for scaling and rotation are fixed at 0.005 and 0.001, respectively. Meanwhile, the degree of spherical harmonics that controls appearance properties is incremented by one level every 1000 iterations. For the native density control mechanism of 3DGS, the algorithm starts densification at the 500-th iteration and ends at the 15,000-th iteration. During this interval, every 100 iterations, the model performs cloning or splitting operations on Gaussian primitives whose view-space positional gradients exceed the predefined threshold $\tau_{\mathrm{grad}}=2\times 10^{-4}$. Additionally, the opacity of all Gaussians is reset to near-zero values every 3000 iterations to remove redundant background components.

The conventional geometric densification in the first 15,000 iterations lays a solid spatial foundation for the subsequent semantic-level feature binding. To address feature aliasing caused by the back-projection of single 2D features under sparse-view conditions, we design a lightweight Joint Feature Encoder. In the feature preprocessing stage, local appearance cues are extracted using a pre-trained ResNet-50 from its layer1 feature maps and are reduced offline to 256-dimensional descriptors. For global semantics, we employ a pre-trained CLIP model with the SwinT-OGC backbone. Input images are resized to a maximum side length of 1333 pixels with the short side fixed at 800 pixels, and the extracted mask-level features are mapped to 512-dimensional semantic vectors. For each 3D Gaussian primitive, we concatenate a 256-dimensional appearance feature, a 512-dimensional semantic feature, and the explicit geometric attributes of 3DGS, namely a 3-dimensional scale vector $s\in R^{3}$ and a 4-dimensional rotation quaternion $q\in R^{4}$, forming a 775-dimensional hybrid feature vector. To reduce the numerical scale discrepancy among heterogeneous modalities, the concatenated vector is first normalized by a LayerNorm layer. It is then passed through a two-layer multilayer perceptron (MLP) with a 512-dimensional hidden layer and GELU activation. This MLP contains parameterized weights as part of the fusion block design, but in the current implementation we do not apply a dedicated mask-consistency loss, standalone training schedule, or external checkpoint to optimize it separately. Instead, the block is used as a lightweight nonlinear projector that compresses the hybrid feature into a unified 512-dimensional Gaussian-level joint representation for subsequent clustering, voting, and global consistency optimization. Its usefulness therefore comes from organizing heterogeneous but already informative cues into a comparable representation, not from learning an independently supervised semantic metric. For this reason, the later cosine-similarity measurements and graph propagation steps should be interpreted as operating on a scene-internal fusion descriptor, rather than on a separately trained semantic metric space.

In the current implementation, the SG-AGS pipeline does not rely on end-to-end joint optimization of SAM, CLIP, RAFT, the ResNet feature extractor, or the Joint Feature Encoder itself. Instead, semantic guidance is introduced through precomputed 2D masks, semantic descriptors, and optical-flow alignment. Gaussian-level semantic labels are obtained by projecting visible Gaussians to the image plane and aggregating the corresponding 2D mask labels through multi-view voting. The resulting vote labels and confidence scores, together with the fused geometry-aware descriptor, are then used in later semantic refinement modules, including decomposition and graph-based consistency propagation, while the underlying reconstruction stage still follows the standard 3DGS photometric objective $L_{\mathrm{rgb}}$. Compared with a post-labeling pipeline on fixed Gaussians, the main additional overhead in the current system comes from four sources: offline RAFT precomputation, cached multi-view semantic/appearance features, local primitive growth after semantic decomposition, and the subsequent k-NN graph optimization.

For feature alignment, we adopt an offline precomputation strategy to avoid prohibitive computational overhead: we use a pre-trained RAFT model [20] with 20 iteration steps to estimate the dense pixel-wise displacement field from all source views to the central reference view. During the feature forward pass, the displacement field is normalized to [−1,1], and PyTorch’s grid-sample function is employed to warp the ResNet feature maps using bilinear interpolation. This design is intended to reduce appearance misalignment before feature fusion under the fixed implementation setting used in this paper; it should not be interpreted as an exhaustive comparison against all possible correspondence formulations such as direct geometric reprojection or depth-aware reprojection.

The semantic-guided adaptive Gaussian decomposition stage [18] is triggered when a Gaussian’s boundary hit rate exceeds $\tau_{\mathrm{bound}}=0.3$ and its multi-view joint voting confidence is lower than $\tau_{\mathrm{conf}}=0.5$. The confidence threshold $\tau_{\mathrm{conf}}=0.5$ corresponds to an uncertain voting state; a voting confidence below this value indicates cross-view label conflict (i.e., no single semantic label has sufficient support), suggesting that the primitive may lie near an instance boundary. Meanwhile, the boundary hit rate threshold $\tau_{\mathrm{bound}}=0.3$ is used to filter accidental projection jitters caused by minor camera pose errors. The hit rate is calculated via multi-view projection onto the 2D SAM mask morphological boundary (dilated with a 3×3 convolution kernel). During splitting, each selected parent Gaussian is replaced by two child Gaussians in the current implementation, which locally increases the number of primitives in decomposition regions. The child Gaussians directly copy the parent’s opacity, rotation, and spherical-harmonic color coefficients as an initialization; no additional opacity renormalization term is introduced at this stage. Their center coordinates are symmetrically offset along the positive and negative directions of the major axis of the 3D covariance, with offset magnitude $\frac{1}{2}\left(1-r\right)L$, where *r* is the semantic split ratio and $L=3\sqrt{\lambda_{\max}}$ is the estimated major-axis extent derived from the leading eigenvalue of the 3D covariance. Their scales are dynamically reduced to 0.6×max(r,0.1) times the original scale, with a lower truncation of $1\times 10^{-6}$. In this way, the scale attenuation is used to partially mitigate the local density increase that may otherwise follow opacity duplication, while the child covariance is re-parameterized from the updated scale and inherited rotation. The Gaussian-level joint features are then recomputed from the updated geometry together with the re-aggregated appearance and semantic cues. This decomposition step is intended to reduce boundary ambiguity while keeping the number of additional primitives localized to uncertain regions. Accordingly, this initialization is interpreted as a practical segmentation-oriented refinement rule rather than a guarantee of exact preservation of the original alpha-compositing behavior, and a dedicated before/after rendering-fidelity evaluation would be needed for separate quantitative characterization. All multi-view masks and CLIP embeddings are cached offline, and a hard-threshold-based noise filtering strategy is supplemented in the post-processing stage to remove redundant background outliers.

#### 4.1.4. Evaluation Metrics

To quantify the segmentation accuracy, we project the extracted 3D Gaussian objects back onto each training viewpoint to generate predicted 2D masks, which are then compared pixel-by-pixel with the manually annotated ground truth from the SPIn-NeRF dataset. As core metrics, we primarily use the mean Intersection over Union (mIoU) and the mean Accuracy (mAcc). The former measures the overlap between predicted and ground-truth masks, while the latter evaluates the model’s recall ability in correctly identifying target pixels [26]. These two metrics quantify overall mask agreement and region-level recognition quality, but they do not isolate boundary fidelity as a separate target. Therefore, in the revised manuscript, claims specifically about boundary refinement are described more cautiously and are supported mainly through the reported visual comparisons of boundary regions rather than presented as a dedicated boundary-metric benchmark. Before metric computation, the predicted masks are binarized and processed with a fixed contour-based cleanup step to suppress small isolated artifacts. In the released evaluation pipeline, frames whose view-wise overlap remains at or below 0.01 are not included in the final mIoU and mAcc averages. Unless otherwise specified, the reported SPIn-NeRF quantitative results correspond to this filtered protocol. We describe this setting explicitly so that the reported comparisons are interpreted under the same stated protocol and subset definition.

While we primarily rely on 2D metrics for quantification, 3D consistency is evaluated through a “Single-Point-Prompt-Multi-View-Verification” protocol. Specifically, we input a single-point prompt at the first viewpoint to initiate segmentation [27], and then evaluate the model’s segmentation performance across all remaining non-interacted viewpoints. If the model maintains a high mIoU score at novel viewpoints, we treat this as indirect evidence that the extracted 3D Gaussian distribution remains relatively coherent across views and shows reduced semantic ambiguity in the reported cases.

### 4.2. Interactive 3D Instance Segmentation

#### 4.2.1. Quantitative Results

In the experimental comparison, we evaluate our method against several representative baselines, including the NeRF-based interactive 3D segmentation method SA3D (Segment Anything in 3D) [28], the 3D Gaussian Splatting-based segmentation method SAGA, and a Single-View baseline that relies solely on single-view observations. SA3D performs prompt-driven segmentation on NeRF representations and highlights the ability of semantic propagation in implicit 3D scene representations. SAGA extends 3D Gaussian Splatting to semantic scene understanding by associating Gaussian primitives with semantic information, thereby providing a strong 3DGS-based baseline for comparison. In contrast, the Single View baseline performs segmentation independently on each view and does not explicitly enforce cross-view consistency. We employ the 2D ground-truth masks provided by the dataset as the supervision signal and use pixel-level Accuracy (Acc) and Intersection over Union (IoU) as the primary evaluation metrics to quantitatively compare the segmentation performance of different methods in realistic sparse-view scenarios.

Table 1 reports mean Intersection over Union (mIoU) and mean Accuracy (mAcc). The table contains eight listed scenes, including Bark as an explicit failure case for SG-AGS. In the current manuscript, the reported average for our method is interpreted with respect to the common seven-scene valid subset defined above, on which our method obtains an mIoU of 92.5% under the stated protocol. Relative to SA3D, the observed gain is consistent with the use of explicit 3D Gaussian primitives and the proposed adaptive decomposition stage, which can reduce boundary blurring caused by mixed primitives in the reported examples. Although SA3D also enables interactive segmentation, its implicit field-based mask back-projection pipeline can suffer from geometric inconsistencies under sparse viewpoints, resulting in fragmented or drifting masks in novel views.

#### 4.2.2. Consistency Check

To further evaluate the stability of segmentation results across viewpoint changes, we designed a consistency verification experiment. In this experiment, we provided a single-point interaction [27] for the first frame of the video sequence. We then rendered segmentation masks for subsequent viewpoints (e.g., frames 10, 20, 30) using the extracted 3D Gaussian model and inspected their boundary stability. In the tested viewpoint changes, the generated masks remain close to the object boundaries, including transitions from front views to side or rear views. Compared with conventional frame-by-frame segmentation methods [29], the presented examples show fewer flickering and local vanishing artifacts. This qualitative behavior is consistent with the intended role of the joint feature aggregation module, which aggregates appearance, semantic, and geometric features in 3D space rather than relying on cues from a single view [11].

#### 4.2.3. Result Analysis

**Figure 3:**
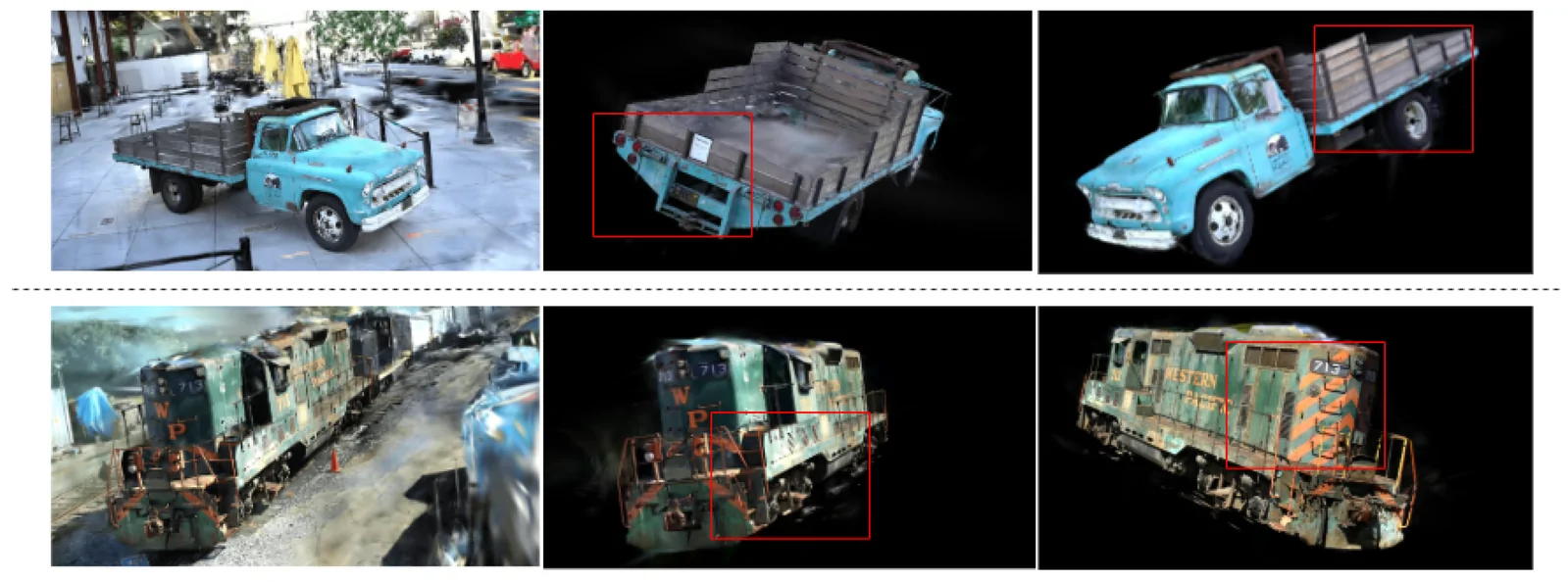
The 2D visualization result of 3D instance segmentation. The red boxes highlight representative boundary regions where local mask continuity and boundary leakage can be compared more clearly.

**Figure 4:**
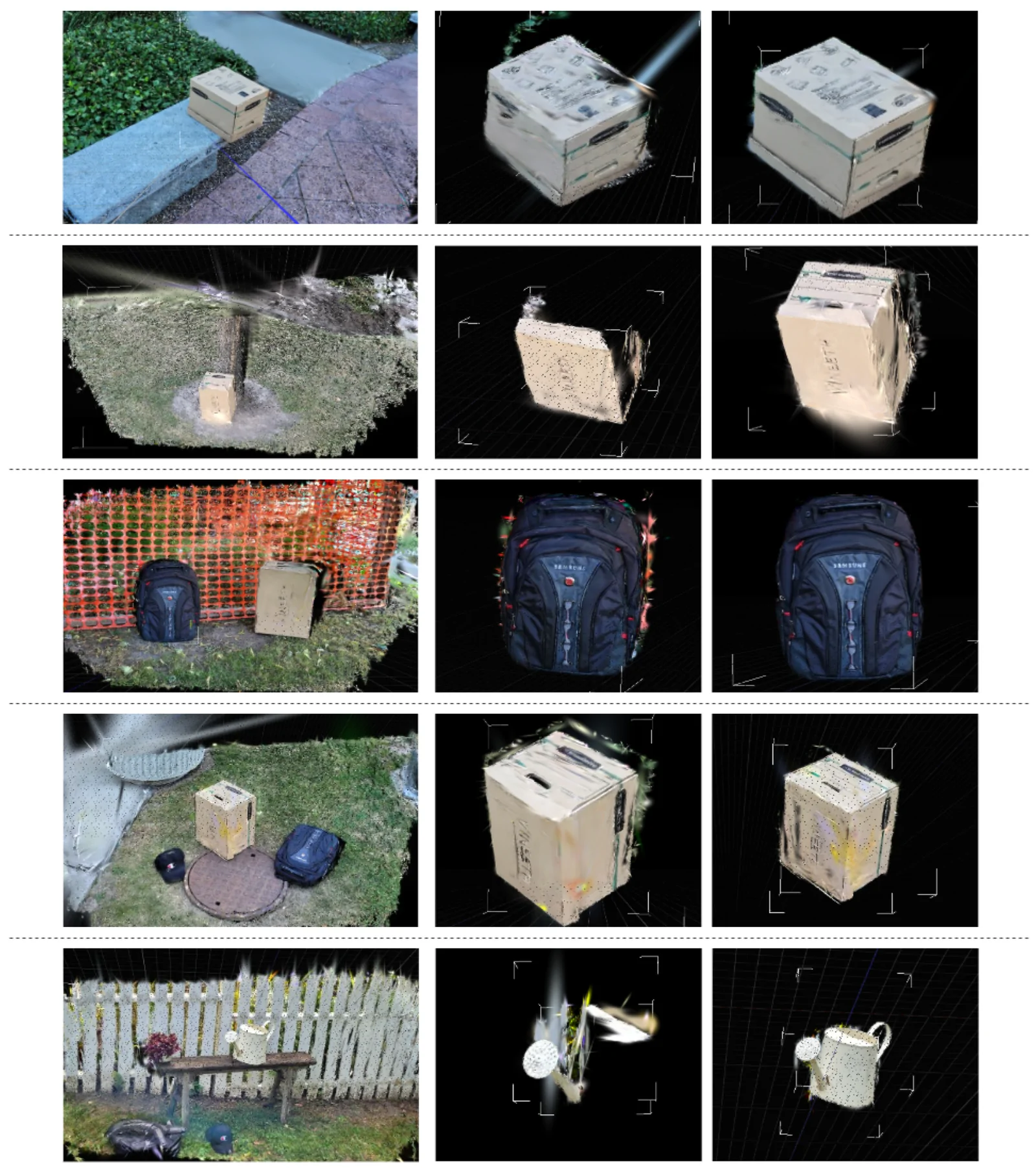
Qualitative results compared with SAGS in different scenes (Spin-NeRF dataset). From left to right: the original reference view, the segmentation results of the SAGS baseline, and the segmentation results of our proposed SG-AGS method. In the reported examples, SG-AGS shows cleaner object boundaries and less background leakage around the segmented regions.

We first qualitatively examine the segmentation behavior at instance boundaries to show how SG-AGS behaves in complicated environments. As illustrated in Figure 3, which contains large-scale objects with detailed geometry such as trucks and locomotives, the predicted masks in our method remain more continuous around several object boundaries. Figure 4 further provides visual comparisons for smaller instances such as water bottles, backpacks, and cartons. In the baseline results, boundary artifacts are visible when target instances touch background structures such as grass, benches, or stairs. For example, the water bottle in Figure 4 and the truck bed in Figure 3 show fragmented edges and background leakage. In the presented examples, SG-AGS shows less of this leakage by selectively decomposing boundary-sensitive Gaussians and thereby reducing mixed foreground-background primitives near object edges. These examples suggest improved local instance separation in the reported cases, while the quantitative benchmark in this manuscript remains based on mIoU and mAcc rather than on a dedicated boundary-specific metric.

Robust 3D segmentation also requires consistency across different views, especially under occlusion. In realistic camera trajectories, an object may be fully visible from one angle but partially hidden from another. Baselines relying on simple majority voting or view-dependent predictions may assign conflicting instance IDs to the same physical object across frames. In 3D renderings, this appears as broken or inconsistent color patches on object surfaces, which indicates label fragmentation. SG-AGS addresses this issue through the k-NN-based global consistency graph described in Section 3.3. When occlusion causes local Gaussian primitives to yield ambiguous, low-confidence voting results, the graph uses spatial proximity and joint-feature similarity to propagate labels from more reliable neighboring primitives [30]. In the tested occluded regions, this strategy is associated with more coherent labels and fewer single-view blind spots.

The observations above are consistent with the benefit of combining geometry with semantics. Indoor and outdoor scenes often contain objects that share similar textures or colors but have different semantic meanings and physical shapes. For example, a dark computer mouse on a dark desk can be difficult for methods that rely mainly on RGB or CLIP features. SG-AGS incorporates explicit Gaussian geometry into the joint feature encoder. The encoder uses rotation quaternions and scale vectors as geometric priors together with appearance and semantic embeddings. Since small objects and planar surfaces often induce different covariance structures, these geometric cues may provide additional information for separating visually similar but physically distinct instances.

However, it is important to acknowledge that SG-AGS is not without limitations and may experience performance degradation under certain extreme conditions. One notable failure case involves highly reflective, transparent, or extensively textureless surfaces, such as mirrors, glass doors, or blank indoor walls. The multi-view feature alignment in our pipeline relies on optical flow, which inherently assumes brightness constancy. Reflective materials violate this assumption, causing severe cross-view alignment errors. Moreover, CLIP-based semantic features are highly susceptible to ambient light interference in these feature-poor regions. Another challenging scenario is dense homogeneous clutter—for instance, a tangled pile of identical cables or overlapping leaves. Because the CLIP-ViT encoder operates at a relatively low feature resolution, tightly packed objects of the same class produce nearly indistinguishable semantic embeddings. In such cases, 2D masks fail to provide reliable gradients under severe mutual occlusion. Without a sharp semantic gradient, the adaptive splitting mechanism remains untriggered, leading the spatial clustering module to mistakenly treat the entire cluster as a single fused entity. The current geometry-aware descriptor and graph refinement can partially regularize local inconsistencies, but they do not yet provide a dedicated uncertainty-aware fallback once the upstream semantic cues themselves collapse. The failed Bark entry in Table 1 is consistent with this broader failure pattern: under sparse-view ambiguity and unstable semantic cues, SG-AGS may fail to produce a valid segmentation result for a scene, which is why we retain Bark explicitly in the scene-wise table instead of folding it into the subset mean. Addressing these edge cases remains an open challenge for future 3D perception research.

### 4.3. Ablation Studies

#### 4.3.1. Effect of Gaussian Decomposition

Table 2 reports the effects of the three main modules in our pipeline. The full model (Case 4) obtains the highest mIoU, while removing individual modules reduces segmentation quality to different degrees. We first examine the effect of semantic guidance.

Datasets such as SPIn-NeRF include challenging geometric layouts, including narrow gaps and thin elongated structures. Under standard 3DGS optimization, some primitives may span physical boundaries and introduce semantic ambiguity near object edges. The semantic-guided splitting mechanism [17] evaluates semantic consistency at both ends of a probing segment defined on the projected 2D covariance, while the actual split is executed along the principal direction of the corresponding 3D covariance. Candidate mixed primitives are then re-parameterized with smaller scales, which improves boundary localization.

As demonstrated by Case 1 in Table 2, removing semantic guidance reduces mIoU to 85.87%. This drop suggests that clustering alone is insufficient when the underlying Gaussian geometry remains mixed across instance boundaries. In practice, the semantic guidance module mainly helps reduce boundary leakage around high-curvature or contacting regions.

#### 4.3.2. Robustness of Multiview Semantic Voting

We next evaluate the multi-view voting mechanism. In sparse-view setups such as SPIn-NeRF, single-view SAM predictions can be affected by occlusion, illumination changes, or motion blur. We use 3D Gaussians as geometric anchors to aggregate semantic labels across all views in which a primitive is visible. Weighted multi-view aggregation reduces the influence of isolated single-frame errors and provides the confidence signal that is later used to suppress semantically unstable decomposition candidates.

Without multi-view voting (Case 2 in Table 2), the overall quantitative metrics decrease from 95.86 to 85.85. This result indicates that aggregating labels across views remains important even when many well-observed regions are already handled reasonably well by the 2D foundation models. Qualitative inspection further shows localized label inconsistencies around partially occluded boundaries where single-view predictions are ambiguous. The voting module therefore contributes mainly to stabilizing labels in these difficult regions.

#### 4.3.3. Feature Alignment Strategy

We also examine the feature alignment strategy. A simple way to build appearance features is to project 3D points back onto 2D images and sample pixel features directly. However, this strategy is sensitive to camera pose errors and sparse-geometry inaccuracies. In regions with repeating patterns or weak textures, such as the side of a truck, small projection errors may sample features from neighboring regions and degrade the joint feature representation.

To solve this, we embedded an explicit optical flow alignment step based on RAFT [20]. Before aggregating anything, we use the RAFT network to map out pixel-level displacement fields between reference and target views, warping the features accordingly. The purpose of this comparison is to verify the contribution of adding appearance alignment within the current pipeline, rather than to rank RAFT against every alternative correspondence strategy.

Removing flow-based alignment (Case 3 in Table 2) introduces more edge artifacts and reduces mIoU from 95.86 to 79.69. This result suggests that the optical flow step helps compensate for projection misalignment before feature aggregation, especially near object boundaries and thin structures. However, this module-level ablation should be read together with the fixed implementation setting in Table 2, rather than as a full sensitivity study over alignment backbones, reprojection strategies, or occlusion-handling variants. Reflective, transparent, or texture-poor regions remain difficult for the current RAFT-based alignment stage, and geometry-aware reprojection, depth-aware correspondence estimation, or confidence-aware feature weighting may provide more stable alternatives in future work.

The ablation in Table 2 is designed to examine the three main modules under a fixed implementation setting. It verifies the roles of feature alignment, multi-view voting, and semantic guidance in the overall system. Other internal settings, including the CLIP-derived semantic cues, geometric attributes in the joint descriptor, the graph-neighbor count *k*, the boundary/confidence thresholds, and the child-Gaussian initialization rule, are kept unchanged across these experiments. Accordingly, this table is intended as a module-level ablation under one fixed system configuration, not as an exhaustive sensitivity analysis over all internal hyperparameters or alignment alternatives. It also does not isolate the contribution of geometric attributes alone from the effect of joint-feature dimensionality or projector capacity, because these internal factors are not varied separately in the current revision. We therefore use this study to discuss the effects of the main modules, while treating those internal settings as implementation choices of the current system rather than as separately isolated ablation factors.

#### 4.3.4. Text-Query-Based Labeling of Segmented 3D Instances

For open-vocabulary understanding, each segmented 3D instance is associated with a 512-dimensional CLIP embedding. In the reported protocol, we first obtain 3D instances and then perform text-query-based category assignment by encoding text prompts into 512-dimensional vectors with the CLIP text encoder and computing cosine similarity against the instance-level embeddings. To keep the evaluation reproducible, we use a fixed prompt template (“a photo of a [CLASS]”) for the ScanNet benchmark, where [CLASS] corresponds to the exact category names defined in the dataset annotations. The predicted category for each 3D instance is determined by the maximum cosine similarity. The same prompt template, category-name alignment, and CLIP text encoder are applied to all evaluated baseline methods under this protocol.

Table 3 and Table 4 report the quantitative results. Under the stated ScanNet protocol, SG-AGS achieves higher mIoU and mAcc than the evaluated Gaussian-based baselines. Methods such as LangSplat and LEGaussians mainly distill 2D features into 3D representations, which can blur boundaries when projected features are noisy. OpenGaussian couples appearance and semantic features with a fixed clustering strategy, which may be less adaptive to varying object scales in indoor scenes. We also compare with MaskClustering, a depth-assisted baseline. Its category-agnostic results are competitive, but its text-query labeling performance in this setting is more sensitive to imperfect 2D masks and low-texture regions.

Furthermore, Table 5 reports text-prompt segmentation results on three SPIn-NeRF scenes as a case study of text-query-based retrieval on segmented 3D instances. Figure 4 provides qualitative comparisons with SAGS and shows that SG-AGS produces cleaner target masks in the reported examples. These SPIn-NeRF results are intended to illustrate text-query usability in representative scenes rather than to serve as a unified benchmark comparable to the ScanNet protocol above.

## 5. Discussion

### 5.1. Interpretation of Results and Working Hypotheses

Our experimental results are consistent with the working hypothesis of this study: geometric ambiguity in Gaussian primitives may be one factor limiting the quality of 3D instance segmentation in 3DGS representations. The segmentation accuracy achieved by SG-AGS, particularly in scenes involving object boundaries and occlusions, suggests that the combination of label allocation and geometry-aware refinement is useful under the evaluated settings.

Specifically, the semantically guided adaptive Gaussian decomposition strategy reduces the under-segmentation phenomenon observed in prior methods. In traditional 3DGS optimization, primitives driven mainly by photometric loss may generate wide, flat Gaussians that span distinct semantic regions, such as table edges and floors. By using semantic gradients to detect these boundary primitives and then carrying out the actual split along the principal direction of the 3D covariance, SG-AGS reduces structural ambiguity before labeling. This observation supports the view that geometry and semantics are coupled in explicit representations. The joint feature encoding module further suggests that incorporating explicit geometric properties, such as scaling and rotation, can provide additional cues for distinguishing spatially adjacent but semantically different instances in the current system. At the same time, the current ablation does not separately control feature dimensionality and projector capacity, so this manuscript does not claim that the observed gain can already be attributed to geometric attributes alone.

### 5.2. Comparison with Previous Studies

From a methodological perspective, the key differences in SG-AGS are not captured by a single module alone but by the way several modules are coupled. Methods that keep geometry fixed and only aggregate 2D labels mainly address semantic assignment after reconstruction, whereas SG-AGS allows boundary-sensitive primitives to be refined before final instance consolidation. At the same time, compared with decomposition-oriented methods whose triggers are dominated by geometric heuristics or local boundary cues, our pipeline uses semantic ambiguity as a diagnosis signal that must be repeatedly supported by cross-view evidence before a split is executed.

More concretely, the mechanism-level distinction of SG-AGS lies in linking decomposition, feature recomputation, and graph propagation into a single semantic verification loop. Semantic confidence is not used here as a standalone label score; it is first used to suppress unreliable decomposition candidates, then the updated primitives receive recomputed joint descriptors, and finally low-confidence labels are regularized through the k-NN graph. Because not every prior work reports results with the same 2D semantic inputs, scene subsets, or evaluation pipeline, our quantitative claims in this manuscript are intentionally limited to the evaluated baselines under the stated protocol rather than presented as a universal ranking against all related Gaussian decomposition methods. Unlike neural field approaches that provide continuous semantics but may lack explicit instance definitions, SG-AGS preserves the discrete primitive structure of 3DGS while adding instance-level semantic organization.

### 5.3. Broader Implications

The SG-AGS framework proposed in this study contributes to 3D scene understanding by producing semantically consistent and geometrically refined 3D instance segmentation under the evaluated settings. The reported examples indicate clearer 3D instance boundaries and more stable semantic-geometric consistency in representative scenes, which may be useful for interactive experiences in extended reality (XR) and digital twin applications, where users need to select and manipulate independent objects within virtual environments. Furthermore, by reducing some misinterpretations caused by visual noise or occlusion in the evaluated scenes, this method can provide more structured information for environmental perception in robotics and intelligent systems. At the current stage, these should be understood as potential application directions inferred from the reported benchmarks rather than as deployment-level validation in broader external environments. These implications should still be interpreted with caution because the current evidence is limited to the reported benchmarks and protocols.

From the perspective of this study itself, the more direct implication is that SG-AGS may support object-level scene understanding in applications where clearer instance boundaries and more stable cross-view association are useful, such as interactive scene selection, digital-twin inspection, and semantic scene querying. At the same time, transferring the reported results to broader real-world environments still requires caution. The current framework depends on the quality of upstream 2D masks, semantic descriptors, and multi-view alignment, and its behavior under more diverse acquisition conditions has not yet been systematically validated beyond the reported benchmarks. In this sense, the present work should be viewed primarily as a study of geometry-semantic coupling in explicit 3D representations, while broader real-world deployment and external-scene validation remain topics for future work.

## 6. Conclusions

This study addresses geometric ambiguity and semantic boundary discontinuity in 3D Gaussian Splatting representations by proposing a semantically guided adaptive segmentation framework. The results show that active geometric refinement is useful for improving 3D instance segmentation, especially near object boundaries and occluded regions. SG-AGS refines ambiguous primitives, builds geometry-aware joint descriptors, and applies multi-view voting with graph-based consistency optimization. Under the reported evaluation protocol and subset definitions, these coupled components show advantages over the evaluated baselines on category-agnostic segmentation and text-query-based open-vocabulary labeling. In the revised manuscript, we therefore describe the contribution of SG-AGS as a mechanism-level integration of semantic candidate diagnosis, verified Gaussian decomposition, and graph-based consistency refinement, rather than as a blanket replacement for all prior boundary-aware Gaussian splitting methods. At the same time, the current evidence is still limited to the evaluated ScanNet and SPIn-NeRF settings, so broader generalization beyond these benchmarks remains to be validated in future work.

## 7. Limitations and Future Work

SG-AGS has several limitations. First, as a semantically guided framework, its final segmentation quality depends on the accuracy of upstream 2D base models such as SAM and CLIP. Our approach corrects some 2D prediction noise through multi-view consistency constraints rather than generating semantics from scratch. Therefore, if the input views contain systematic 2D segmentation errors or if CLIP features are ambiguous for rare categories [31], these biases may propagate into the 3D representation. Second, transparent or highly reflective objects can violate the brightness constancy assumption used by RAFT-based optical flow, producing misaligned appearance features and unreliable edge weights in the k-NN graph. Under severe occlusion or large viewpoint changes, the same appearance-alignment stage can also become less reliable because valid cross-view correspondences become sparse or unstable. Third, for densely packed homogeneous objects or textureless regions, 2D semantic masks can become unstable. Although the decomposition stage uses boundary hit rate and low-confidence verification to suppress many unreliable triggers, this safeguard is not perfect. Under severe mutual occlusion, heavy projection ambiguity, or strongly viewpoint-dependent artifacts, the labels sampled at the endpoints of the $3\sqrt{\lambda_{1}}$ probing segment may still become unreliable, causing either unnecessary Gaussian splits or missed splits between visually similar instances. In addition, our decomposition rule deliberately separates the 2D semantic diagnosis axis from the 3D split axis: the former is used to detect boundary inconsistency and determine the sign after projection, while the latter is defined by the 3D covariance itself. This avoids directly equating 2D and 3D principal directions, but it does not fully remove the instability caused by perspective distortion. When a Gaussian becomes nearly isotropic, when its leading eigenvalues are close, or when the projected 3D principal axis is severely shortened in a particular view, the sign estimation derived from the 2D diagnostic can still become unreliable. Because the current implementation does not include an explicit eigen-gap rejection rule or isotropy-aware fallback strategy, such cases remain a genuine limitation of the present method rather than a resolved issue. In addition, the present candidate-selection stage does not explicitly model the full projected Gaussian footprint, semantic overlap area, or alpha contribution during the initial boundary pre-screening. For reflective, transparent, or texture-poor regions, more geometry-aware or depth-aware correspondence schemes, together with confidence-aware feature weighting, may further improve robustness. For densely packed homogeneous objects, stronger instance-level descriptors or an explicit uncertainty-aware fallback mechanism may also help when upstream CLIP cues become ambiguous, but these components are not yet included in the current system. The present ablation study also focuses on the three top-level modules under one fixed implementation setting, so it does not yet provide a controlled modality-level comparison that isolates the contribution of geometric attributes from feature dimensionality or projector capacity alone. More broadly, the current experimental evidence is still limited to the two evaluated benchmarks, namely ScanNet and SPIn-NeRF. Although these datasets provide complementary indoor and sparse-view settings, they do not yet cover a wider range of external captures, larger outdoor environments, dynamic scenes, or more diverse acquisition conditions. For this reason, broader generalization beyond the reported benchmarks should still be regarded as an open question for future validation. Finally, part of the SPIn-NeRF quantitative comparison depends on two additional conditions: the reported filtered view-wise protocol excludes extremely low-overlap frames from the final averages, and the summary statistics for SG-AGS are interpreted on the common seven-scene valid subset after the Bark failure case is retained separately. The corresponding quantitative gains should therefore be read together with these dataset-specific reporting conditions.

A further limitation is that the introduction of active geometric refinement mechanisms inevitably increases computational overhead. Multi-view feature aggregation, gradient-based adaptive Gaussian splitting (SAGD), and k-NN global consistency optimization all involve computationally intensive processes whose complexity scales with the number of Gaussian primitives. In the current implementation, the use of offline optical-flow precomputation and cached multi-view masks and semantic embeddings also introduces nontrivial storage and memory overhead. While this trade-off yields higher segmentation accuracy and boundary quality, current implementations remain better suited for offline reconstruction and analysis tasks rather than real-time applications compared to methods relying solely on post-processing labeling. In addition, because decomposition duplicates opacity as an initialization and increases the number of primitives locally, the rendering-quality effect of splitting deserves a more dedicated evaluation. A more complete analysis of before/after Gaussian counts, preprocessing time, graph-optimization time, GPU memory usage, feature-storage overhead, rendering FPS, PSNR, SSIM, LPIPS, and transmittance changes would help isolate these trade-offs quantitatively and remains important future work. Future work could alleviate this computational bottleneck by simplifying candidate verification or introducing more efficient sparse graph propagation algorithms.

Building upon the current SG-AGS framework, several future directions remain open. One possible direction is to combine the segmented scene representation with semantically aware scene editing. Since our method separates scenes into geometrically independent instances with semantic labels, it can provide a starting point for object-level manipulation [32]. Future work could explore the use of 3D generative models to repaint or replace textures on selected segmented instances, such as transforming a wooden chair into a metal chair, while preserving the surrounding environment.

Another direction is large language model-driven scene interaction. By treating segmented 3D instances as a structured “visual vocabulary”, SG-AGS could be connected with multimodal large language models for natural-language-based scene querying. Furthermore, extending the adaptive decomposition strategy to dynamic scenes is another possible direction for future work. If such extensions become practical, they may broaden the use of explicit 3D representations in robotics and autonomous driving domains.

## Figures and Tables

**Table 1:** Quantitative comparison of 3D semantic segmentation across different methods.

Scenes	Single View		SA3D		SAGA		Ours	
	IoU	Acc	IoU	Acc	IoU	Acc	IoU	Acc
Otters	75.4	96.0	83.6	96.9	85.4	97.5	85.9	99.5
Ferns	93.2	96.3	83.2	96.6	86.5	98.9	95.9	99.7
Room	85.4	90.5	85.3	98.3	86.6	98.1	93.6	99.3
Fork	73.3	96.3	94.5	99.0	91.1	98.6	92.9	98.9
Fortress	94.1	97.1	89.3	99.8	95.6	99.3	94.2	99.6
Bark	69.4	88.5	80.4	99.6	83.4	99.3	-	-
Truck	69.0	85.2	90.8	99.1	83.0	97.9	94.1	99.7
Lego	73.9	77.9	96.7	99.1	90.0	97.9	90.9	99.1
Mean	80.6	91.3	89.1	98.4	88.3	98.3	92.5	99.4
Std	10.2	7.3	5.3	1.2	4.2	0.6	3.3	0.3

**Table 2:** Performance ablation on different proposed modules for the 3D instance segmentation task.

Case	Feature Alignment	Multi-Perspective Voting	Semantic Guidance	mIoU	mAcc
#1	√	√		85.87	99.54
#2	√		√	85.85	99.53
#3		√	√	79.69	97.60
#4	√	√	√	95.86	99.53

*Note*: √ indicates that the corresponding module is included in the experiment.

**Table 3:** Performance of category-agnostic 3D instance segmentation on the Scannet dataset.

Type	Methods	mIoU	mAcc
Depth	MaskClustering	54.17	80.95
Gaussian-based	GaussianGrouping	22.55	30.54
	OpenGaussian	27.32	52.44
	InstanceGaussian	50.27	80.22
	SG-AGS (ours)	78.50	96.41

**Table 4:** Performance of text-query-based open-vocabulary labeling on segmented 3D instances in the ScanNet dataset.

Type	Methods	mIoU	mAcc
Depth	MaskClustering	38.10	56.74
Gaussian-based	LangSplat	8.40	22.06
	LEGaussian	12.82	28.62
	OpenGaussian	38.29	55.19
	InstanceGaussian	47.94	64.01
	SG-AGS (ours)	77.49	89.63

**Table 5:** Quantitative segmentation results on three SPIn-NeRF scenes using text prompts.

Scene	Box	Backpack	Garden
Text	“the box”	“the backpack”	“the watering can”
IoU	84.20	82.93	75.87
Acc	89.63	89.28	89.53

## Data Availability

The public datasets used for model training and evaluation are openly available in the community. The experimental results data generated in this study are available on request from the corresponding author.
